# Supplementary material for: Genomic epidemiology of the first two waves of SARS-CoV-2 in Canada
Source: eLife. 2022 Aug 2;11:e73896. doi: 10.7554/eLife.73896 (PMC9345601; doi:10.7554/eLife.73896)
Supplement: Supplementary file 3. [file elife-73896-supp3.pdf]

Supplementary File 3. Supplemental tables from subsampling sensitivity analysis

McLaughlin, A., Montoya, V., Miller, R. L., Mordecai, G. J., Canadian COVID-19 Genomics Network Consortium (CanCOGen), Worobey, M., Poon, A. F. Y., & Joy, J. B. (2022). Genomic epidemiology of the first two waves of SARS-CoV-2 in Canada. eLife. https://doi.org/10.7554/eLife.73896

Supplementary File 3a. Number of Canadian and global sequences available and subsampled in the four subsampling strategies, where 25-100% of Canadian sequences were retained.

Month	n Canadian sequences sampled				n Canadian sequences available	n global sequences sampled				n global sequences available
	25%	50%	75%	100%		25%	50%	75%	100%	
2019-12	0	0	0	0	0	21	21	21	21	21
2020-01	5	5	5	5	5	465	465	465	465	465
2020-02	15	15	15	15	15	1360	1360	1360	983	1360
2020-03	774	1735	3186	3347	3347	3248	2483	1717	983	44447
2020-04	774	1735	3186	4473	4473	3248	2483	1717	983	44634
2020-05	774	1735	1821	1821	1821	3248	2482	1717	983	22810
2020-06	761	761	761	761	761	3248	2482	1717	983	25879
2020-07	667	667	667	667	667	3248	2482	1717	983	29702
2020-08	774	1358	1358	1358	1358	3248	2482	1717	983	33172
2020-09	774	1684	1684	1684	1684	3247	2482	1717	983	34353
2020-10	774	1735	2127	2127	2127	3247	2482	1717	983	56902
2020-11	773	1735	3186	4240	4240	3247	2482	1717	983	80464
2020-12	773	1735	3186	3585	3585	3247	2482	1717	983	111794
2021-01	773	1734	3185	6125	6125	3247	2482	1716	983	189984
2021-02	773	1734	3185	6528	6528	3247	2482	1716	982	217196
**TOTAL**	9184	18368	27552	36736	36736	40816	31632	22448	13264	893183

Supplementary File 3b. Total number of cases, sequences available, and sequences sampled by region and by subsampling strategy with 25-100% Canadian sequences retained in a representative bootstrap.

Region	n cases	n clean seq. available	n seq. 25%	n seq. 50%	n seq. 75%	n seq. 100%
USA	28747597	231535	13369	9721	6360	3367
Europe	16786160	179488	4138	3131	2282	1345
India	11112241	11217	3613	3058	2192	1236
Brazil	10558091	6278	2672	2313	1780	1122
Asia	9058898	56922	2918	2342	1783	1134
Latin America	7404854	6020	1696	1367	1001	591
Russia	4198400	2569	1301	1021	758	480
United Kingdom	4188823	290323	1765	1349	935	489
France	3815688	12898	1581	1144	827	511
Spain	3188553	19382	1766	1311	862	432
Italy	2925292	9744	1406	1147	836	573
Africa	2567496	7630	549	400	293	173
Germany	2442336	30446	1088	829	562	340
Mexico	2086938	3806	785	640	438	250
Iran	1631169	129	95	91	91	74
South Africa	1513393	5173	725	523	332	184
Peru	1323863	1225	518	427	326	193
China	109017	936	757	753	743	745
Oceania	32717	17274	74	64	47	25
Ontario	300813	12450	3577	6637	10011	12450
Quebec	287740	7710	3052	5306	7303	7710
Alberta	129347	4342	646	1438	2369	4342
British Columbia	80454	9376	1388	3906	6137	9376
Manitoba	31859	1235	420	732	1055	1235
Saskatchewan	14244	193	38	114	162	193
Maritimes	4058	1430	63	235	515	1430
**TOTAL**	114540041	929731	50000	49999	50000	50000

Supplementary File 3c. For each of the four subsampling strategies with 25-100% of Canadian data subsampled, ten bootstrap datasets were summarized by the number of tips pruned from the initial maximum likelihood trees as temporal outliers based on having residuals further than the mean +/- 3 standard deviations of residuals in the linear regression of evolutionary divergence over time, or based on having more than 12 mutations on the pendant edge (final branch) leading to the tip. The slope of the strict clock rate models before and after removing outliers are reported with linear model R^2 values.

Subsampling strategy	Boot-strap	Initial n tips	Initial strict clock rate (s/s/y)	Initial R^2	Tips excl., residuals	Tips excl., pendant edge	Final strict clock rate (s/s/y)	Final R^2	Final n tips
25%	1	50,000	9.21E-04	0.564	1851	683	7.40E-04	0.676	47,466
25%	2	50,000	8.84E-04	0.589	590	712	8.37E-04	0.672	48,698
25%	3	50,000	8.53E-04	0.650	266	663	8.37E-04	0.689	49,071
25%	4	50,000	7.92E-04	0.370	1093	688	6.77E-04	0.381	48,219
25%	5	50,000	9.12E-04	0.584	1279	689	8.04E-04	0.638	48,032
25%	6	50,000	7.28E-04	0.472	369	678	7.03E-04	0.501	48,953
25%	7	50,000	1.05E-03	0.443	1941	686	7.37E-04	0.629	47,373
25%	8	50,000	7.89E-04	0.565	94	704	7.81E-04	0.661	49,202
25%	9	50,000	8.37E-04	0.552	260	677	8.19E-04	0.674	49,063
25%	10	50,000	8.14E-04	0.651	468	638	7.83E-04	0.696	48,894
50%	1	50,000	8.30E-04	0.641	636	542	7.85E-04	0.687	48,822
50%	2	50,000	8.59E-04	0.563	645	529	8.02E-04	0.667	48,826
50%	3	50,000	8.84E-04	0.579	1514	504	7.38E-04	0.684	47,982
50%	4	50,000	8.28E-04	0.579	727	518	7.70E-04	0.661	48,755
50%	5	50,000	8.34E-04	0.575	106	515	8.25E-04	0.689	49,379
50%	6	50,000	8.56E-04	0.570	1435	522	7.16E-04	0.656	48,043
50%	7	50,000	6.83E-04	0.428	112	567	6.75E-04	0.529	49,321
50%	8	50,000	7.47E-04	0.486	432	494	7.16E-04	0.502	49,074
50%	9	50,000	8.85E-04	0.580	1426	535	7.47E-04	0.689	48,039
50%	10	50,000	7.68E-04	0.528	1130	572	6.74E-04	0.577	48,298
75%	1	50,000	7.56E-04	0.667	318	402	7.36E-04	0.700	49,280
75%	2	50,000	7.88E-04	0.621	899	408	7.17E-04	0.684	48,693
75%	3	50,000	7.54E-04	0.653	363	414	7.31E-04	0.694	49,223
75%	4	50,000	8.24E-04	0.596	1249	452	7.09E-04	0.689	48,299
75%	5	50,000	7.73E-04	0.646	127	381	7.66E-04	0.698	49,492
75%	6	50,000	8.02E-04	0.641	1015	436	7.22E-04	0.706	48,549
75%	7	50,000	7.05E-04	0.484	464	399	6.70E-04	0.533	49,137
75%	8	50,000	7.58E-04	0.627	420	428	7.28E-04	0.692	49,152
75%	9	50,000	7.91E-04	0.655	400	396	7.64E-04	0.696	49,204
75%	10	50,000	7.99E-04	0.436	1460	382	6.12E-04	0.538	48,158
100%	1	50,000	7.53E-04	0.616	417	345	7.27E-04	0.665	49,238
100%	2	50,000	7.49E-04	0.663	175	409	7.40E-04	0.691	49,416
100%	3	50,000	7.22E-04	0.653	85	371	7.17E-04	0.675	49,544
100%	4	50,000	7.43E-04	0.684	22	389	7.43E-04	0.704	49,589
100%	5	50,000	7.69E-04	0.603	760	380	7.09E-04	0.656	48,860
100%	6	50,000	7.25E-04	0.579	223	374	7.08E-04	0.666	49,403
100%	7	50,000	7.40E-04	0.650	270	387	7.24E-04	0.683	49,343
100%	8	50,000	7.40E-04	0.653	169	373	7.30E-04	0.693	49,458
100%	9	50,000	7.83E-04	0.610	42	375	7.78E-04	0.686	49,583
100%	10	50,000	7.22E-04	0.646	72	386	7.19E-04	0.690	49,542
**Mean for each strategy**									
25%	Mean	50,000	8.58E-04	0.544	821.1	681.8	7.72E-04	0.622	48,497
50%	Mean	50,000	8.17E-04	0.553	816.3	529.8	7.45E-04	0.634	48,654
75%	Mean	50,000	7.75E-04	0.603	671.5	409.8	7.16E-04	0.663	48,919
100%	Mean	50,000	7.45E-04	0.636	223.5	378.9	7.30E-04	0.681	49,398

Supplementary File 3d. For each of the four subsampling strategies with 25-100% of Canadian data subsampled, and for each of ten bootstrap datasets, maximum likelihood trees were time-scaled using LSD2 in IQTREE-2 under a relaxed log normal clock with 0.2 relative variance. The mean and 95% confidence interval (CI) of the mean relaxed clock rate and the time of most recent common ancestor (TMRCA) are reported and summarized by strategy.

		Strict clock rate	Relaxed clock rate			TMRCA		
**Strategy**	**Bootstrap**		**Mean**	**Lower 95% CI**	**Upper 95% CI**	**Mean**	**Lower 95% CI**	**Upper 95% CI**
25%	1	7.40E-04	2.81E-04	2.77E-04	2.85E-04	2019-12-23	2019-12-03	2019-12-24
25%	2	8.37E-04	2.91E-04	2.86E-04	2.94E-04	2019-12-24	2019-11-28	2019-12-24
25%	3	8.37E-04	2.87E-04	2.81E-04	2.90E-04	2019-12-24	2019-11-19	2019-12-24
25%	4	6.77E-04	2.97E-04	2.94E-04	3.01E-04	2019-12-24	2019-12-01	2019-12-24
25%	5	8.04E-04	NA	NA	NA	NA	NA	NA
25%	6	7.03E-04	2.89E-04	2.85E-04	2.94E-04	2019-12-24	2019-11-30	2019-12-24
25%	7	7.37E-04	2.86E-04	2.81E-04	2.90E-04	2019-12-23	2019-11-29	2019-12-24
25%	8	7.81E-04	2.86E-04	2.82E-04	2.89E-04	2019-12-24	2019-11-28	2019-12-24
25%	9	8.19E-04	2.90E-04	2.85E-04	2.94E-04	2019-12-24	2019-12-06	2019-12-24
25%	10	7.83E-04	2.89E-04	2.85E-04	2.93E-04	2019-12-24	2019-12-01	2019-12-24
50%	1	7.85E-04	2.92E-04	2.87E-04	2.97E-04	2019-12-23	2019-11-29	2019-12-24
50%	2	8.02E-04	2.89E-04	2.86E-04	2.95E-04	2019-12-24	2019-12-04	2019-12-24
50%	3	7.38E-04	2.85E-04	2.82E-04	2.91E-04	2019-12-24	2019-11-26	2019-12-24
50%	4	7.70E-04	2.94E-04	2.91E-04	2.97E-04	2019-12-24	2019-12-05	2019-12-24
50%	5	8.25E-04	2.88E-04	2.84E-04	2.93E-04	2019-12-24	2019-12-06	2019-12-24
50%	6	7.16E-04	2.91E-04	2.88E-04	2.95E-04	2019-12-23	2019-12-01	2019-12-24
50%	7	6.75E-04	2.84E-04	2.80E-04	2.88E-04	2019-12-22	2019-12-01	2019-12-22
50%	8	7.16E-04	2.97E-04	2.95E-04	3.03E-04	2019-12-24	2019-12-03	2019-12-24
50%	9	7.47E-04	2.97E-04	2.94E-04	3.03E-04	2019-12-24	2019-12-02	2019-12-24
50%	10	6.74E-04	2.95E-04	2.92E-04	3.01E-04	2019-12-24	2019-11-30	2019-12-24
75%	1	7.36E-04	2.80E-04	2.78E-04	2.88E-04	2019-12-20	2019-11-18	2019-12-22
75%	2	7.17E-04	2.87E-04	2.84E-04	2.93E-04	2019-12-23	2019-12-03	2019-12-24
75%	3	7.31E-04	2.82E-04	2.81E-04	2.89E-04	2019-12-21	2019-11-25	2019-12-23
75%	4	7.09E-04	2.91E-04	2.89E-04	2.98E-04	2019-12-24	2019-12-01	2019-12-24
75%	5	7.66E-04	2.84E-04	2.81E-04	2.90E-04	2019-12-22	2019-11-27	2019-12-24
75%	6	7.22E-04	2.90E-04	2.88E-04	2.97E-04	2019-12-23	2019-11-30	2019-12-24
75%	7	6.70E-04	2.82E-04	2.79E-04	2.87E-04	2019-12-21	2019-11-09	2019-12-22
75%	8	7.28E-04	2.85E-04	2.83E-04	2.90E-04	2019-12-23	2019-11-16	2019-12-24
75%	9	7.64E-04	2.86E-04	2.83E-04	2.92E-04	2019-12-22	2019-11-27	2019-12-23
75%	10	6.12E-04	2.94E-04	2.92E-04	3.00E-04	2019-12-23	2019-11-27	2019-12-24
100%	1	7.27E-04	2.79E-04	2.77E-04	2.88E-04	2019-12-15	2019-11-14	2019-12-18
100%	2	7.40E-04	2.92E-04	2.89E-04	3.02E-04	2019-12-17	2019-10-23	2019-12-19
100%	3	7.17E-04	2.85E-04	2.84E-04	2.95E-04	2019-12-17	2019-11-19	2019-12-19
100%	4	7.43E-04	2.84E-04	2.82E-04	2.93E-04	2019-12-16	2019-11-21	2019-12-19
100%	5	7.09E-04	2.84E-04	2.84E-04	2.93E-04	2019-12-15	2019-10-26	2019-12-18
100%	6	7.08E-04	2.69E-04	2.69E-04	2.77E-04	2019-12-14	2019-11-12	2019-12-16
100%	7	7.24E-04	2.96E-04	2.95E-04	3.04E-04	2019-12-19	2019-11-25	2019-12-22
100%	8	7.30E-04	2.65E-04	2.64E-04	2.73E-04	2019-12-15	2019-11-10	2019-12-17
100%	9	7.78E-04	2.65E-04	2.64E-04	2.72E-04	2019-12-14	2019-11-24	2019-12-17
100%	10	7.19E-04	2.62E-04	2.62E-04	2.70E-04	2019-12-14	2019-11-23	2019-12-17
**Mean for each strategy**								
25%	Mean	7.72E-04	2.89E-04	2.84E-04	2.92E-04	2019-12-24	2019-11-29	2019-12-24
50%	Mean	7.45E-04	2.91E-04	2.88E-04	2.96E-04	2019-12-24	2019-12-01	2019-12-24
75%	Mean	7.15E-04	2.86E-04	2.84E-04	2.92E-04	2019-12-22	2019-11-24	2019-12-23
100%	Mean	7.30E-04	2.78E-04	2.77E-04	2.87E-04	2019-12-17	2019-11-13	2019-12-19
